# Basic Hand Gestures Classification Based on Surface Electromyography

**DOI:** 10.1155/2016/6481282

**Published:** 2016-05-19

**Authors:** Aleksander Palkowski, Grzegorz Redlarski

**Affiliations:** Department of Mechatronics and High Voltage Engineering, Gdańsk University of Technology, Ulica G. Narutowicza 11/12, 80-233 Gdańsk, Poland

## Abstract

This paper presents an innovative classification system for hand gestures using 2-channel surface electromyography analysis. The system developed uses the Support Vector Machine classifier, for which the kernel function and parameter optimisation are conducted additionally by the Cuckoo Search swarm algorithm. The system developed is compared with standard Support Vector Machine classifiers with various kernel functions. The average classification rate of 98.12% has been achieved for the proposed method.

## 1. Introduction

Surface electromyography (SEMG) is a noninvasive method of measurement of the bioelectrical activity of muscles. SEMG is used both in the diagnosis of diseases of the muscular system and in the development of man-machine interfaces. However, the accuracy of systems that translate human muscle activity into the motion of various machinery or accuracy of sophisticated analysis of human motion based on SEMG signals is still an open problem.

Several successful systems for EMG classification can be pointed out. Examples of systems previously developed include the use of the *k*-Nearest Neighbors algorithm in combination with Bayesian analysis, which achieves correct classification rate of 94% [1]. Better results are achieved with techniques that use neurofuzzy adaptive inference (95% of correctly classified samples) [2], wavelet analysis (97.4%) [3], or a combination of wavelet analysis with the Support Vector Machine classifier (95%) [4]. Even better results are achieved when the Support Vector Machine classifier is subjected to additional parameter optimisation process done by the Particle Swarm Optimisation algorithm (97.41% of correctly classified samples) [5].

As it is apparent from the exemplary works presented above, there is still room for improvement, and a question is raised as to what modification should be introduced to achieve possibly even better results. This paper presents a novel classification system for SEMG signals based on the Support Vector Machine (SVM) classifier optimised by the Cuckoo Search (CS) algorithm. The SEMG signals are derived from measurements of the flexor carpi radialis and extensor carpi radialis longus muscles and are subjected beforehand to proper parametrisation in the time domain. The system was used to classify six basic hand gestures: hand closing, hand opening, wrist flexion, wrist extension, index finger straightening, and thumb straightening. As a result, a rate of 98.12% of correctly classified samples was achieved.

## 2. Methods

### 2.1. Acquisition and Segmentation of the SEMG Signal

The SEMG signal was measured using the NeuroTrac MyoPlus2 device from two locations on the forearm of a healthy man—the flexor carpi radialis and extensor carpi radialis longus muscles. Measurements were made at 16 Hz.

**Figure 1:**
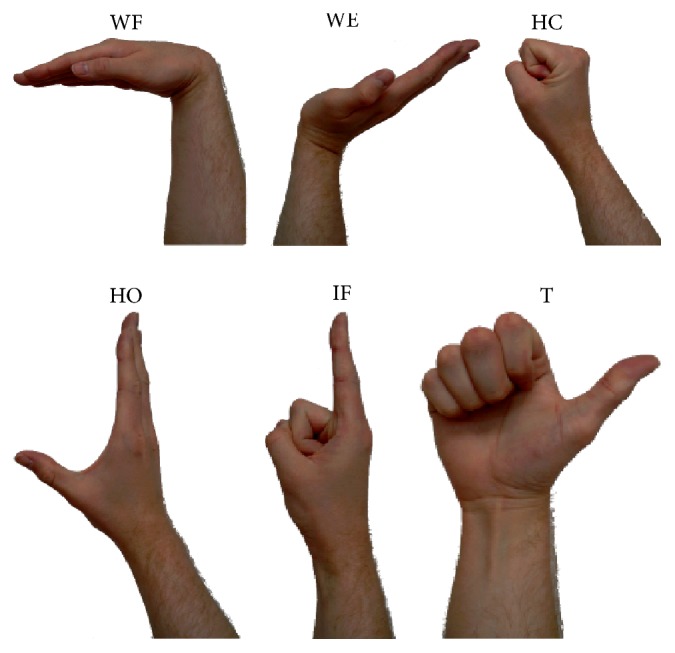
The hand gestures tested.

**Figure 2:**
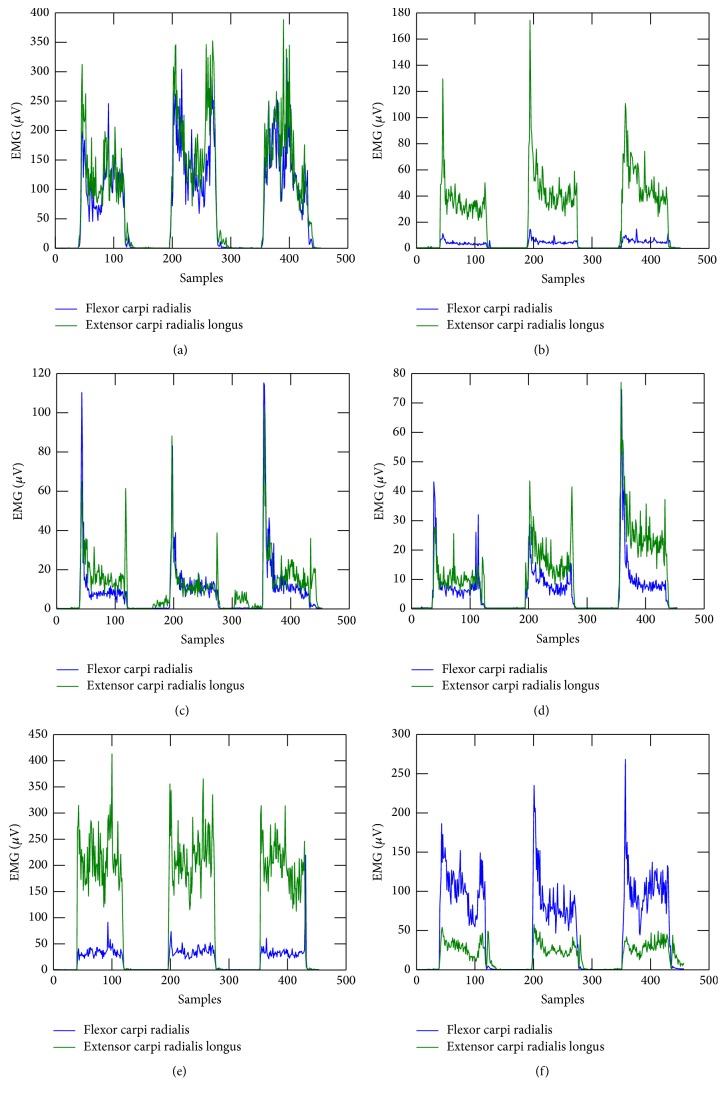
Examples of SEMG signals measured. (a) Hand closing. (b) Hand opening. (c) Index finger straightening. (d) Thumb straightening. (e) Wrist extension. (f) Wrist flexion.

The SEMG signal was measured while performing six types of hand gestures: hand closing (HC), hand opening (HO), wrist flexion (WF), wrist extension (WE), index finger straightening (IF), and thumb straightening (T).  Figure 1 presents the hand gestures performed. Each set of gestures consisted of twenty samples performed as a sudden change of hand position from a relaxation state to one of the above gestures and then holding the gesture for five seconds, followed by return to the relaxation state. Examples of SEMG signals collected are presented in Figure 2.

Proper segmentation of the SEMG signal is important in case of building systems that should react to the movement of the body. Segments that are too short lead to falsification of results, whereas too long segments cause unnecessary computational load when analysing signals in real time. There are two types of segmentation which can be applied to the SEMG signals: sliding segmentation and overlapping segmentation. An SEMG signal segment of about 200 ms contains enough information to properly classify it, and at the same time it is so short that the use of sliding segmentation does not negatively affect the operation of the system [6]. However, in the case of segments longer than 200 ms, it is recommended to use the overlapping segmentation [7]. During the research described, the sliding segmentation with a single segment of length 187.5 ms was used.

### 2.2. SEMG Signal Feature Extraction

Selection of an appropriate data representation has a significant impact on the effectiveness of a classifier. SEMG signals can be described through three types of representation: in the time, frequency, time-frequency, or time-scale domain [7, 8]. The time-scale domain is not recommended for the analysis of SEMG signals because of the large computational load it puts on the calculations and the need to further reduce the size of the feature set [9]. Moreover, the use of transformations in the frequency domain is also associated with unnecessary computational load and does not provide significantly better results in the classification process [6, 9, 10]. Therefore, the most commonly used representations of the SEMG signals are those in the time domain.

The following features can be distinguished in particular among the transformations in the time domain: mean absolute value (MAV), waveform length (WL), Willison amplitude (WAMP), and slope sign change (SSC). The literature proves their high efficiency in supporting the classification process, particularly when using a single coefficient (MAV or WL) or their combinations (MAV, WL, and WAMP or MAV, WL, and SSC) [6, 9]. Formulae for the aforementioned features for a set of signal data points *x*_*i*_,  *i* ∈ {1,2,…, *N*}, can be found below:

(1) MAV=1N∑i=1Nxi,

(2) WL=∑i=1N−1xi+1−xi,

(3) WAMP=∑i=1N−1fxi−xi+1,

(4) SSC=∑i=2N−1fxi−xi−1×xi−xi+1,

A series of tests were performed to determine the best feature (or set of features) to be used for the case described. The results of these tests are presented in Section 3.

### 2.3. Support Vector Machine Classifier

The Support Vector Machine (SVM) is a binary supervised classifier, the aim of which is to determine the optimal hyperplane that separates two groups (classes) of points in space [11, 12]. The hyperplane must meet the requirement of having a maximum margin, that is, being maximally distant from both classes.

The problem presented is nonlinear; therefore, in order to deal with nonlinearity of the feature space particular kernel functions are used. The most commonly used functions are quadratic, polynomial, and radial basis functions.

Although originally the SVM was created as a binary classifier, there are strategies, the use of which extends the capabilities of the SVM to classify multiple classes simultaneously. The strategies that can be used are one against all, everyone against everyone, and all at the same time [13]. The strategy applied in the research reported is one against all, in which each class is separated and compared to the others, thereby forming a number of classifiers equal to the number of classes analysed. The choice of the strategy is dictated by its ease of implementation, speed, and relatively high efficiency in comparison with the other strategies mentioned [6].

### 2.4. Cuckoo Search Optimisation

The use of the SVM as a classifier yields good results, especially with respect to electromyographic signal [4, 6]. However, there still remains the problem of proper selection of the kernel function and the parameters of the classifier, which—tuned to a particular case—may help in the calculation of an even better adjusted hyperplane. This problem is nontrivial, so the common motion is to use metaheuristic methods to solve it.

A group of metaheuristic optimisation methods, which are gaining increased recognition, are the swarm algorithms. Swarm algorithms are based on the concept of distributed intelligence, according to which relatively low-complex actions of many agents and their mutual interaction lead to a new and better quality. Swarm algorithms are used in a wide range of optimisation problems [14]. The literature shows several examples of successful application of swarm optimisation to tune the SVM classifier. Furthermore, electromyographic signal classifiers benefit from this combination of methods as their classification rate is higher than other methods compared [5].

One of the most prominent swarm algorithms is the Cuckoo Search (CS) algorithm, developed by Yang and Deb [15]. The CS algorithm is inspired by the brood parasitism phenomenon seen in some species of cuckoos, which lay their eggs in nests of birds of other species. The algorithm applies the mechanism of Lévy flights to select subsequent nests, which allows for proper balance between exploration and exploitation of the search space. The main assumptions of the algorithm are as follows:

- Each cuckoo lays one or more eggs (in a randomly chosen nest) that represent coordinates of a point in the search space, being the problem solution.
- Some nests with the best value of fitness function are moved to the next iteration.
- The number of nests is fixed and at the end of each iteration a part of them is rejected with some probability.

Based on the above presented assumptions, an optimisation algorithm was developed. Brief description of the CS algorithms is presented in Algorithm 1.

The CS algorithm proved to be efficient in several optimisation tasks [14, 16]. Moreover, the CS algorithm used as a means of choosing appropriate kernel function and parameters for the SVM classifier proved to be improving further the classifier's capabilities, outperforming even the artificial neural network classifier [17].

### 2.5. Optimisation Procedure for the SVM

The CS algorithm used in the study was designed to optimise the coefficients of the SVM classifier and choose the optimal kernel function for a given class. The choice of the kernel function was made from the quadratic, polynomial, and radial basis functions.

**Figure 3:**
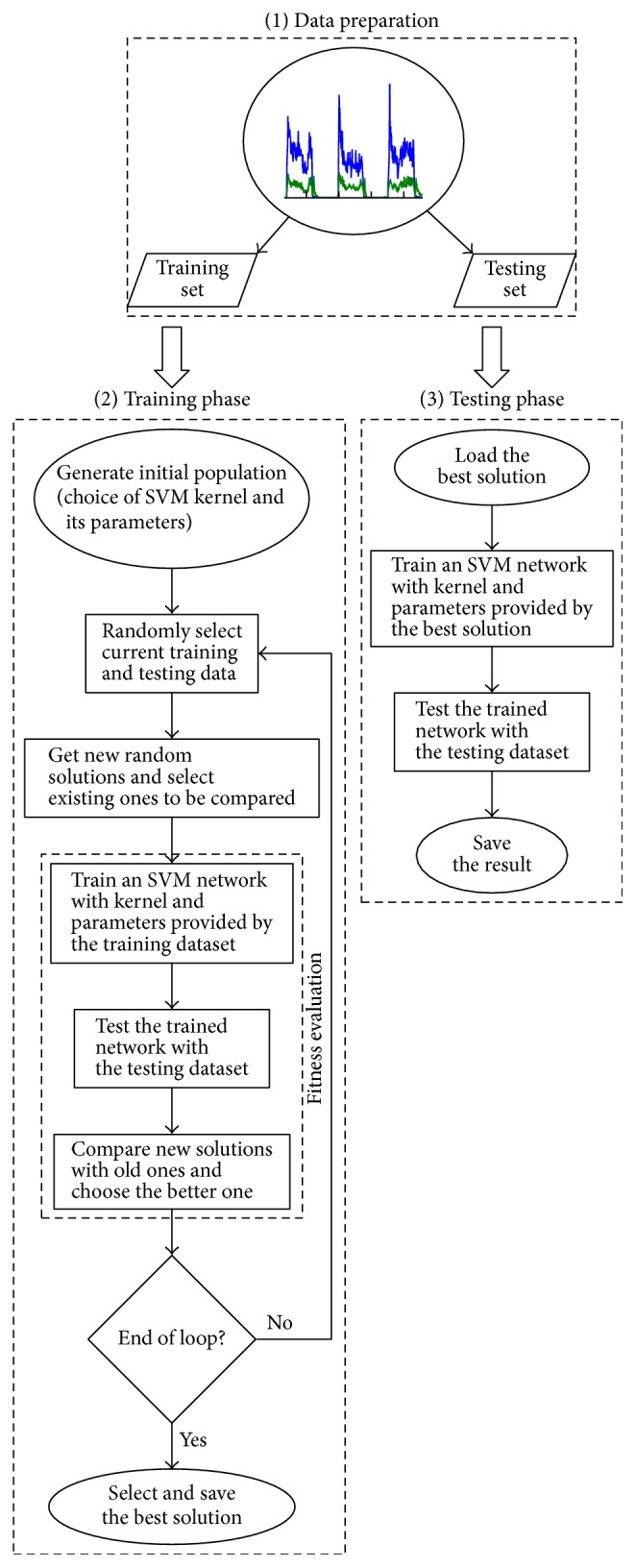
Schematic of the experimentation procedure.

Figure 3 presents a generalised experimentation procedure scheme and the procedure is as follows:

1. The whole dataset is divided into the main training and testing sets.
2. The CS algorithm iterations start. Each cuckoo contains a solution, that is, the SVM kernel function and its parameters, coded as integers (the kernels) and real numbers (the parameters). Every fitness function evaluation, an SVM network is trained using the coded solution and randomly chosen training set and then the network is tested with the other part of the main training dataset, thus providing a classification rate.
3. After performing the CS optimisation, the best solution found is used as the SVM parameters for classifying the testing set (i.e., generalising), thus providing the final result.

Therefore, the optimisation algorithm uses the correct classification rate of the training set during 10-fold cross-validation as its fitness function. If the same result was obtained, each solution was compared further by the number of support vectors.

## 3. Results and Discussion

The study was divided into two parts. In the first part, the quality of classification by using the SVM with various kernel functions was cross-examined with several sets of features that describe the bioelectrical activity of muscles obtained (Tables 1, 2, and 3). In the second part, the SVM-CS classifier developed was tested using the best feature set chosen from the previous tests (Table 4). The SEMG measurements collected were divided into learning and testing groups, making every time a random selection of samples.

Based on the classification results collected for different features that describe the SEMG signal, it can be stated that the best of them is the characterisation made on the basis of the mean absolute value of the signal. The average correct classification rate was above 96% for each kernel function tested. However, it can be assumed that this result was affected by the small number of SEMG channels being considered and the small range of hand gestures performed. If a more complex study was performed using SEMG multichannel motion detection, the information that the other features provide may be useful. In this case, the introduction of unnecessary amount of information for the classifier probably resulted in its overfitting. It should be also noted that there is no single kernel function, for which the results are clearly the best.

The results of the SVM-CS classification system developed show that the additional optimisation of SVM parameters positively affects its efficiency. In certain cases (wrist flexion and extension), the results did not exceed what was previously shown. However, the overall average correct classification rate of 98.12% distinguishes the method presented.

Positive effects of the optimisation algorithm can be also seen in the number of support vectors created during the training process. Their average number per class was 5.01, which compared to the best result of the classifier without additional optimisation (22.1) is a major improvement. It should be also mentioned that the average standard deviation of the results obtained by the SVM-SC classifier amounted to 0.004, which is more than an order of magnitude smaller than the smallest standard deviation obtained with the SVM classifier without optimisation. This again confirms the reliability of the SVM-SC classifier presented.

## 4. Conclusions

The paper presents a novel classification system developed to distinguish six types of hand gestures based on surface electromyographic signals acquired from the flexor carpi radialis and extensor carpi radialis longus muscles. The system presented is built upon the Support Vector Machine classifier and the Cuckoo Search optimisation algorithm. The purpose of the Cuckoo Search algorithm is to choose appropriate kernel function and its parameters.

The system developed is compared with a standard Support Vector Machine classifier. The average correct classification rate of the new system is 98.12%, which is better by 1.31% compared to the best result obtained by the Support Vector Machine classifier without additional optimisation. Moreover, judging by the standard deviation of the results, the optimised classifier is a more reliable solution for the problem presented.

It is also shown that with the set of signals provided the best feature to distinguish hand movements is the mean absolute value of the SEMG signal.

Due to the use of the Cuckoo Search algorithm as an additional optimiser for the classifier's parameters, this paper presents the first such method applied to the SEMG signal analysis.

## Figures and Tables

**Algorithm 1:**
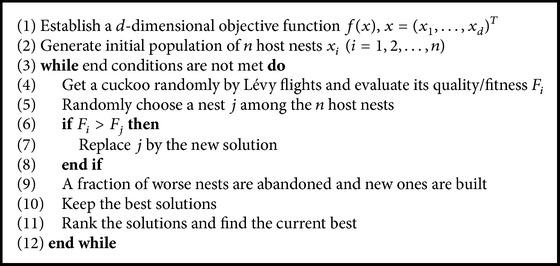
Cuckoo Search algorithm.

**Table 1:** Percent of correctly classified samples by the Support Vector Machine with the quadratic kernel.

Features used	HC	HO	WF	WE	IF	T	Mean
MAV	97.01	94.01	99.32	99.21	96.93	92.8	96.58
WL	85.01	83.13	89.89	88.3	84.14	82.73	85.53
MAV + WL + WAMP	92.16	87.88	98.51	98.06	93.89	90.2	93.45
MAV + WL + SSC	91.67	85.13	98.04	97.79	92.36	88.54	92.26

**Table 2:** Percent of correctly classified samples by the Support Vector Machine with the polynomial kernel.

Features used	HC	HO	WF	WE	IF	T	Mean
MAV	96.8	94.28	99.52	99.01	97.81	93.36	96.81
WL	83.65	81.32	89.67	87.25	85.01	81.8	84.78
MAV + WL + WAMP	89.39	86.83	98.67	97.72	93.27	89.8	92.61
MAV + WL + SSC	86.82	82.58	97.87	97.54	91.61	86.38	90.47

**Table 3:** Percent of correctly classified samples by the Support Vector Machine with the radial basis function kernel.

Features used	HC	HO	WF	WE	IF	T	Mean
MAV	97.3	94.6	98.83	98.61	97.97	93.47	96.8
WL	84.36	83.43	88.98	87.73	85.09	83.34	85.49
MAV + WL + WAMP	89.58	88.42	95.21	95.17	94.92	92.76	92.68
MAV + WL + SSC	90.9	86.03	98.24	97.82	92.28	87.94	92.2

**Table 4:** Percent of correctly classified samples by the Support Vector Machine optimised by the Cuckoo Search algorithm.

HC	HO	WF	WE	IF	T	Mean
97.97	96.53	99.56	99.33	98.12	97.22	98.12
